# Mouse-adapted H9N2 avian influenza virus causes systemic infection in mice

**DOI:** 10.1186/s12985-019-1227-4

**Published:** 2019-11-12

**Authors:** Zhe Hu, Yiran Zhang, Zhen Wang, Jingjing Wang, Qi Tong, Mingyang Wang, Honglei Sun, Juan Pu, Changqing Liu, Jinhua Liu, Yipeng Sun

**Affiliations:** 1 Key Laboratory of Animal Epidemiology of the Ministry of Agriculture, College of Veterinary Medicine, China Agricultural University, Beijing, 100193 China; 2 Beijing Huadu Yukou Poultry Industry Co. Ltd., Beijing, 101206 China

**Keywords:** H9N2 avian influenza virus, Adaption, Mutation, Mice

## Abstract

**Background:**

H9N2 influenza viruses continuously circulate in multiple avian species and are repeatedly transmitted to humans, posing a significant threat to public health. To investigate the adaptation ability of H9N2 avian influenza viruses (AIVs) to mammals and the mutations related to the host switch events, we serially passaged in mice two H9N2 viruses of different HA lineages — A/Quail/Hong Kong/G1/97 (G1) of the G1-like lineage and A/chicken/Shandong/ZB/2007 (ZB) of the BJ/94-like lineage —and generated two mouse-adapted H9N2 viruses (G1-MA and ZB-MA) that possessed significantly higher virulence than the wide-type viruses.

**Finding:**

ZB-MA replicated systemically in mice. Genomic sequence alignment revealed 10 amino acid mutations coded by 4 different gene segments (PB2, PA, HA, and M) in G1-MA compared with the G1 virus and 23 amino acid mutations in 5 gene segments (PB1, PA, HA, M, and NS) in ZB-MA compared to ZB virus, indicating that the mutations in the polymerase, HA, M, and NS genes play critical roles in the adaptation of H9N2 AIVs to mammals, especially, the mutations of M1-Q198H and M1-A239T were shared in G1-MA and ZB-MA viruses. Additionally, several substitutions showed a higher frequency in human influenza viruses compared with avian viruses.

**Conclusions:**

Different lineages of H9N2 could adapt well in mice and some viruses could gain the ability to replicate systemically and become neurovirulent. Thus, it is essential to pay attention to the mammalian adaptive evolution of the H9N2 virus.

## Main text

H9N2 avian influenza viruses (AIVs) have been circulating in multiple avian species and are repeatedly transmitted to mammals, including humans and pigs [1–3]. In addition, since H9N2 viruses cause mild infections in humans, causing a typical human flu-like illness that can easily go unnoticed, they have the opportunity to adapt to humans. Recent evidence suggested that H9N2 viruses isolated from China after 2010 have displayed higher virulence to chicken and mice, and some naturally isolated H9N2 viruses tested were transmissible in ferrets by respiratory droplets [4]. In 2013, a novel reassortant H7N9 virus carrying six internal genes from H9N2 AIV caused serious outbreaks in humans in China [5–7]. In fact, the susceptibility of H7N9 to humans is thought to be partly due to the adaptability of H9N2 to mammals [8–10]. Thus, investigating the adaptation of H9N2 to mammals will be helpful for the prevention of avian influenza virus interspecies transmission. Phylogenetic analysis indicated that the HA genes of H9N2 viruses in China mainly fall into two lineages, the BJ/94-like and G1-like [1, 3]. Human infections with these two lineages were often observed [11]. However, research on the adaptive genetic basis of G1-like and BJ/94-like H9N2 influenza viruses to mammals is still scarce. Mice are ideal animal models for investigating the pathogenic mechanisms and host range determinants of influenza virus, and they can be used to generate mouse-adapted variants by serial lung-to-lung passages [12, 13]. Some studies showed that BJ/94-like viruses could adapt well to mice [12, 14]. However, there has been no research on the adaptation of G1-like H9N2 viruses to mice. In the present study, we generated two mouse-adapted H9N2 viruses by serial lung passages and studied the pathogenicity and molecular variation of the two mouse adaptive viruses. We found that the G1-like H9N2 strain adapted more rapidly than the BJ/94-like virus and that ZB-MA virus gained the ability to replicate systemically and became neurovirulent. These results provide a theoretical basis to evaluate the potential threat of H9N2 to public health.

**Fig. 1:**
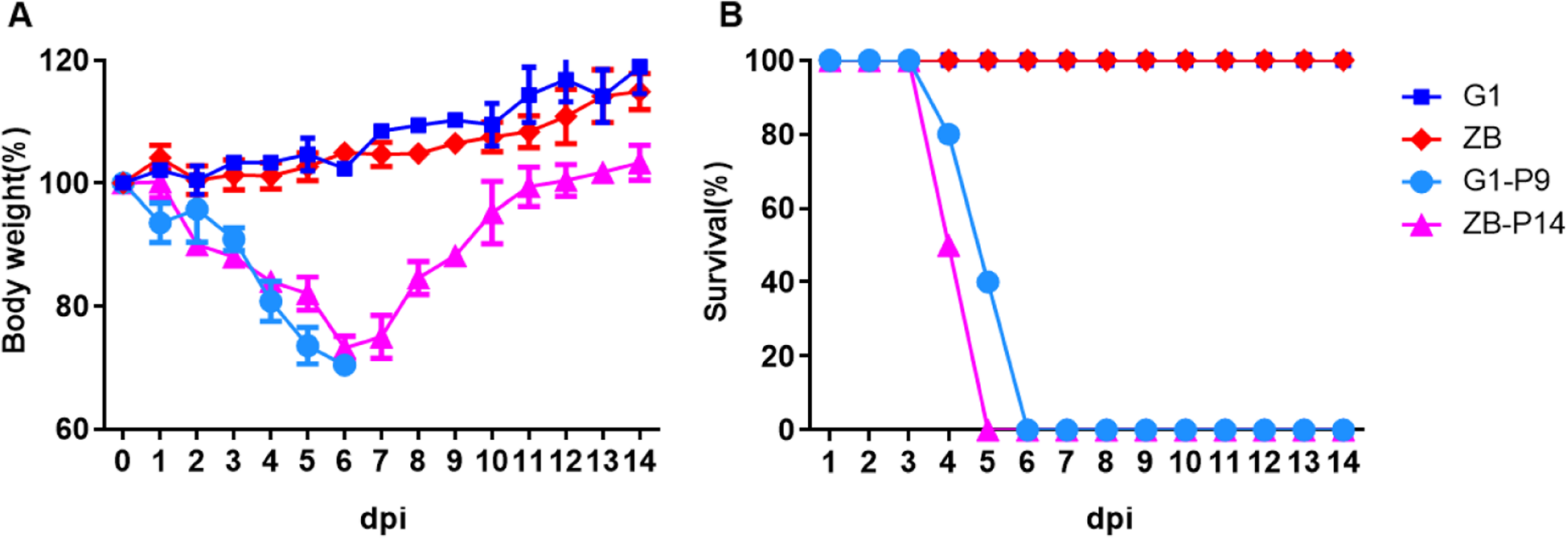
Virulence of G-P9, ZB-P14, and wild-type viruses in mice. Groups of five six-week-old BALB/c mice were inoculated intranasally with G1-P9, ZB-P14, or wild-type H9N2 viruses. Body weight (**a**) and survival (**b**) were monitored daily for 14d

To generate mouse-adapted viruses, the G1 strain and ZB strains were serially passaged in six-week-old female mouse lungs, beginning with an intranasal inoculation of 10^5^ pfu of virus per mouse. At 3 days post-inoculation (dpi), mice were killed, their lungs were harvested and homogenized, and 50 μl of supernatant from the centrifuged homogenate were used as inoculum for the next passage. Survival and clinical symptoms of infected animals were recorded after each passage. The wild-type G1 and ZB were almost avirulent in mice, and the weight loss of the infected mice became more and more serious with the increase in the number of generations of the virus. After nine passages, all of the mice inoculated in the G1 virus died at 6 dpi (Fig.1), whereas after 14 passages of the ZB virus all inoculated mice died at 5 dpi.

**Fig. 2:**
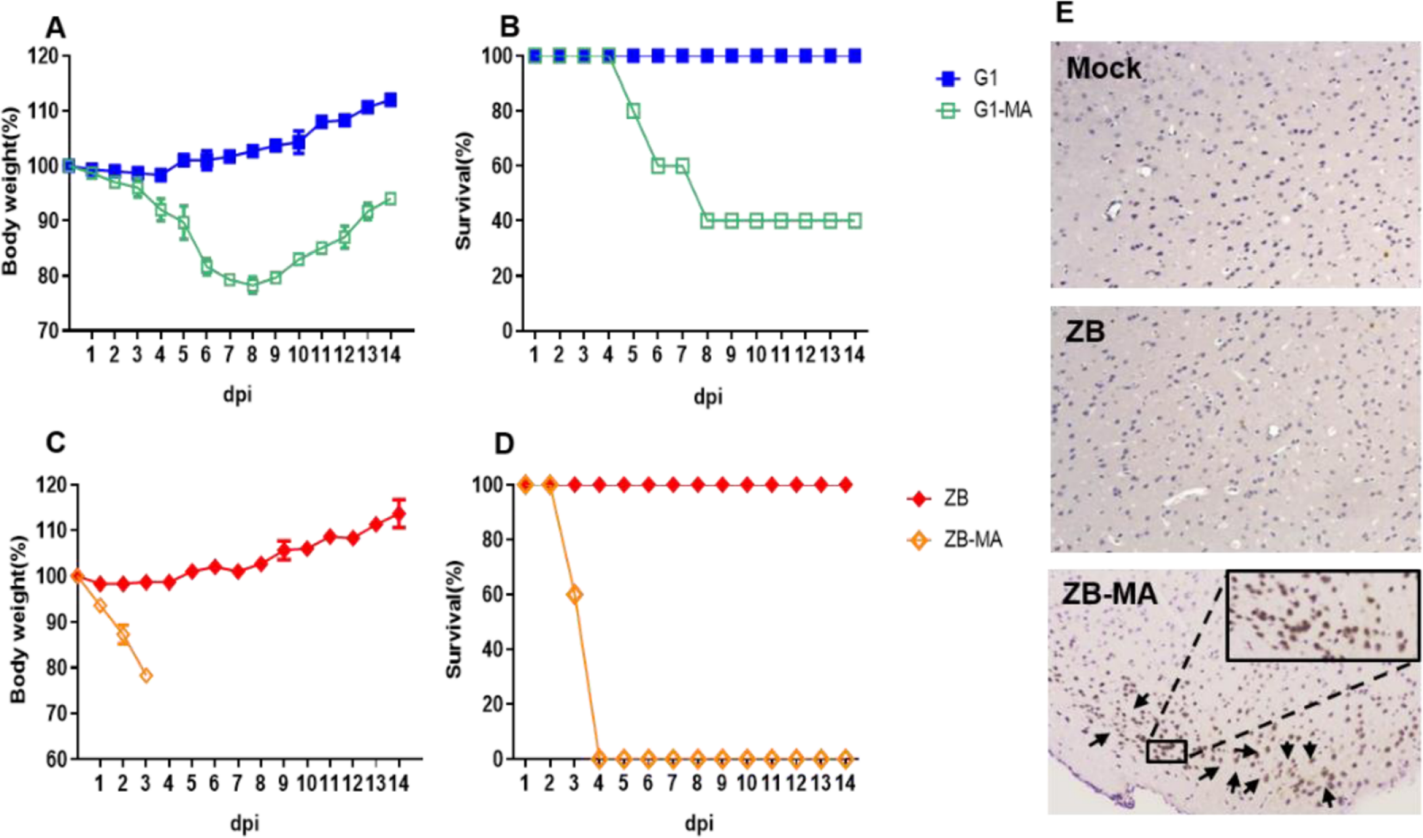
Virulence of G1-MA, ZB-MA and wild-type viruses in mice. **a** and **c** Relative weight loss. Mice were intranasally inoculated with 10 ^5^ pfu of each virus. The body weights of five inoculated mice were measured daily and are represented as the percentage of the weight on the day of inoculation (day 0). **b** and **d** Survival percentages of mice infected with 10^5^ pfu of each virus. **e** Immunohistochemically-stained of the brains Groups of eight six-week-old BALB/c mice were inoculated intranasally with 10^5^ pfu of each viruses. IHC examinations were performed on the cerebrum of mice inoculated with PBS, ZB or ZB-MA virus. Sections of brains of mice infected with indicated viruses at 5 dpi are shown. In the immunohistochemical-stained brain sections, the viral antigen-positive cells are marked by brown pigment. Arrows show that cell positive for influenza virus signals were detected in the brains of infected mice. Images were taken at × 400 magnification

To further study the pathogenicity of mouse adaptive strains to mammals, we performed plaque purification and genome sequencing to the acquired monoclonal strains of G1 P9 and ZB P14 (named G1-MA and ZB-MA respectively). Next, we compared the virulence of the generated mouse-adapted variant with that of the wild-type strains. The MLD_50_ value showed that G1-MA was > 10^2.15^-fold more virulent than G1 virus and ZB-MA was > 10^2.75^-fold more virulent than ZB virus (Table 1). All of the mice infected with 10^5^ pfu of G1-MA or ZB-MA exhibited severe clinical signs of disease, including decreased activity, huddling, hunched posture, and ruffled fur. Mice in the G1-MA group began to die at 5 dpi (Fig. 2 a and b), and mice in the ZB-MA group began to die at 3 dpi (Fig. 2 c and d). In contrast, the parental mice infected with 10^5^ pfu of wild type virus displayed weight increase over a period of 14 days and no morbidity or mortality was observed. To determine whether the differences in virulence of the mouse-adapted H9N2 and wild-type strains were due to a different ability to spread and replicate in the mouse organs, groups of three BALB/c mice were killed at 3 dpi, and various organs, including the liver, spleen, lung, kidney, and brain, were harvested for virus detection and titration. G1-MA and ZB-MA replicated to significantly high levels in the lungs, reaching viral titers 10^1.3^-fold and 10^2.0^-fold higher than those of G1 virus and ZB, respectively (*p* < 0.05). The ZB-MA virus was also detected in liver, spleen, lung, kidney and brain (Table 2 and Fig.2e), indicating that the ZB-MA virus had gained the ability to replicate in all of the tested organs (systematically), including the central nervous system. No infectious virus was detected in any organ other than the lung from any of the mice infected with any of the G1, G1-MA, and ZB viruses.

**Table 1:** Virulence change of G1 and ZB virus during mouse adaption

Virus	MLD_50_ (log_10_EID_50_ml^− 1^)	Change in MLD_50_ (log_10_decrease)
G1	> 6	NA^a^
ZB	> 6	NA
G1-MA	3.85	> 2.15
ZB-MA	3.25	> 2.75

^a^ Not applicable

**Table 2:** Viral distribution of wild-type and mouse-adapted viruses in different tissues in mice

Virus	Mean virus titer in sample [log_10_ TCID_50_ ml^−1^] ± SD				
	Lung	Liver	Spleen	Kidney	Brain
G1	3/3^a^	-^b^	–	–	–
	(5.6 ± 0.6)				
G1-MA	3/3	–	–	–	–
	(6.9 ± 0.3^c^)				
ZB	3/3	–	–	–	–
	(5.0 ± 0.1)				
ZB-MA	3/3	2/3	3/3	2/3	3/3
	(7.0 ± 0.1^d^)	(2.6 ± 0.1)	(3.3 ± 0.1)	(3.1 ± 0.2)	(3.7 ± 0.2)

^a^No. of infected mice/total no. of mice

^b^No virus was isolated from the sample

^c^The lung titers in the G1-MA group were significantly higher than those in the G1 group (*p* < 0.05)

^d^The lung titers in the ZB-MA group were significantly higher than those in the ZB group (*p* < 0.05)

**Fig. 3:**
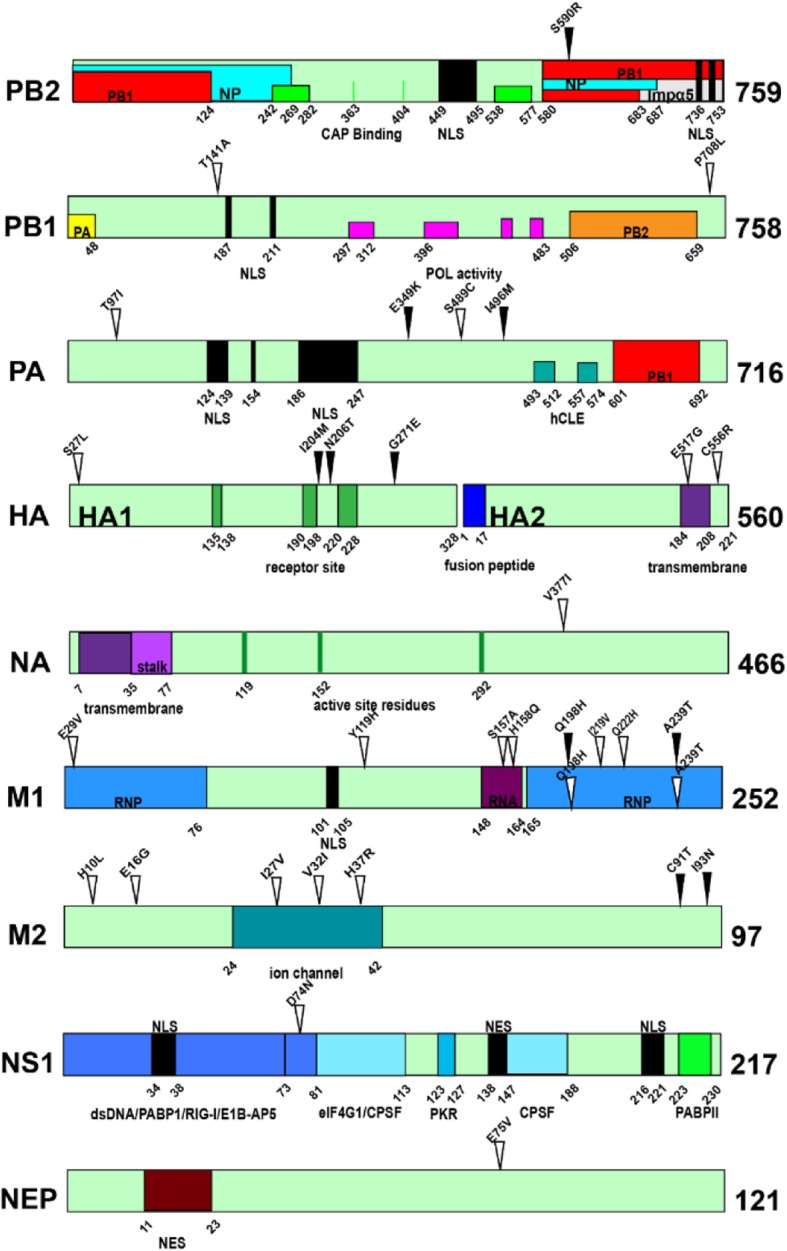
Schematic diagram of influenza viral protein function. The amino acid location of mutations in G1-MA are numbered and indicated with solid triangle on the linear sequence and mutations in ZB-MA are numbered and indicated with hollow triangle. The locations of regions of protein binding, or functions, are indicated with rectangles and are labeled with respect to the interacting viral proteins

The pathogenesis of influenza A viruses is a polygenic trait and understanding the molecular mechanisms important to the host range and pathogenesis is critical for the prevention and treatment of influenza A virus infections [15]. To identify the molecular basis for the enhanced virulence acquired by H9N2 viruses during adaption, the genomes of the wild-type and mouse-adapted viruses were sequenced. The comparison of the full coding sequences between mice adapted strains and their corresponding wild-type strains revealed 10 and 23 amino acid mutations in G1-MA and ZB-MA, respectively (Table 3 and Table 4). M1-Q198H and M1-A239T were found in both G1-MA and ZB-MA viruses and 97% of human H3N2 influenza viruses possessed M1-239 T. Except for M1-239 T, there were 9 additional substitutions in ZB-MA strain also presented more frequently in human H9N2, pH1N1 or human H3N2 influenza viruses, including PA-S489C, NA-V377I, M1-S157A, M1-H158Q, M1-Q222H, M2-H10L, M2-E16G, M2-I27V, and M2-V32I. Additionally, PA-T97I, PA-E349K and HA-I204M have also been detected in mice-adapted strains previously [16–18]. These results indicated the related substitutions might be important for the mammalian adaptation of H9N2 influenza viruses. In order to analyze the possible molecular basis of these mutations leading to increased pathogenicity, we further analyzed the functional areas where these mutations are located (Fig. 3). In the G1-MA virus, HA-I204M and HA-N206 T occur in the receptor binding site and PB2-S509R is located in the overlapping regions of the NP and PB1 binding domains; M1-Q198H and M1-A239T reside in the RNP binding region. In the ZB-MA virus, the M1-E29V, M1-Q198H, M1-I219V, M1-Q222H, and M1-A239T mutations reside in the RNP binding region, M1-S157A and M1-H158Q mutations reside in RNA binding region; the M2-I27V, M2-V32I, and M2-H37R mutations occur in the ion channel.

**Table 3:** Amino acid differences between the wild-type strain G1 and the mouse-adapted strain G1-MA

Proteins	AA site	G1	G1-MA	Avian H9N2	Human H9N2	pH1N1	Human H3N2
PB2	590^a^	S	R	G (97.5%)	S (100%)	S (85%)	T (47%)
							S (45%)
							G (8%)
						G(15%)	
				S (2.5%)			
PA	349^c^	E	K	E (100%)	E (100%)	E (100%)	E (100%)
	486	I	M	I (100%)	I (100%)	I (100%)	I (100%)
HA	204 (196)^b c^	I	M	T (95%)	T(60%)	/	/
				K (4.2%)	I (40%)		
				A (0.6%)			
				I (0.2%)			
	206 (198)	N	T	T (95%)	T (60%)	/	/
				A (4.2%)			
				N (0.8%)			
					N (40%)		
	271 (263)	G	E	E (99%)	E (60%)	/	/
				G (0.5%)			
				V (0.5%)	G (40%)		
M1	198	Q	H	Q (100%)	Q (100%)	Q (99%)	Q (100%)
						H (1%)	
	239	A	T	A (100%)	A (100%)	A (100%)	**T (97%)**^d^
							A (2%)
							V (1%)
M2	91	C	F	F (99.8%)	F (100%)	F (51%)	F (100%)
						T (47%)	
						V (1%)	
				C (0.2%)		L (1%)	
	93	I	N	N (99.5%)	N (100%)	N (89%)	S (99%)
				I (0.1%)			
				K (0.1%)			
				S (0.1%)			
				T (0.1%)		S (9%)	
						D (1%)	N (1%)
						T (1%)	

^a^H9 numbering

^b^H3 numbering is in parentheses

^c^The substitutions also occurred in mice-adapted influenza viruses in the previous studies

^d^The substitution which presents more frequently in human influenza viruses than H9N2 AIVs was shown by bold and underline formats

**Table 4:** Amino acid differences between the wild-type strain ZB and the mouse-adapted strain ZB-MA

Proteins	AA site	ZB	ZB-MA	Avian H9N2	Human H9N2	pH1N1	Human H3N2
PB1	141^a^	T	A	T (100%)	T (100%)	T (100%)	T (100%)
	708	P	L	P (100%)	P (100%)	P (100%)	P (100%)
PA	97^c^	T	I	T (100%)	T (100%)	T (99%)	T (100%)
						N(1%)	
	489	S	C	C (97%)	**C (100%)**^d^	**C (100%)**	**C (100%)**
				S(3%)			
HA	27 (19)^b^	S	L	S (100%)	S (100%)	/	/
	517 (508)	E	G	E (99%)	E (100%)	/	/
				D (1%)			
	556 (544)	C	R	C (100%)	C (100%)	/	/
NA	377	V	I	V (89%) I (11%)	V (44%) **I (56%)**	/	V (48%)
							T (47%)
							I (5%)
M1	29	E	V	E (100%)	E (100%)	E (100%)	E (100%)
	119	Y	H	Y (100%)	Y (100%)	Y (100%)	Y (100%)
	157	S	A	A (89%)	**A (100%)**	S (100%)	S (100%)
				S (11%)			
	158	H	Q	Q (94%)	**Q (100%)**	**Q (100%)**	**Q (100%)**
				H (6%)			
	198	Q	H	Q (100%)	Q (100%)	Q (99%) H (1%)	Q (100%)
	219	I	V	V (87%)	V (40%)	I (100%)	I (100%)
				I (13%)	I (60%)		
	222	Q	H	H (88%)	**H (100%)**	**H (100%)**	**H (100%)**
				Q (12%)			
	239	A	T	A (100%)	A (100%)	A (100%)	**T (97%)** A (2%)
M2	10	H	L	L (57.1%)	**L (100%)**	P (98%)	P (100%)
				P (29.1%)		H (1%)	
				H (13.0%)			
				R (0.7%)		L (1%)	
				A (0.1%)			
				I (0.1%)			
				Y (0.1%)			
	16	E	G	E (85%)	**G (99%)**	E (87%)	**G (99%)**
				V (12%)		G (13%)	E (1%)
				G (3%)			
	27	I	V	V (92%)	**V (100%)**	**V (100%)**	**V (97%)**
				I (5%)			I (1%)
				M (3%)			T (1%)
							A (1%)
	32	V	I	I (83%)	**I (100%)**	**I (100%)**	**I (100%)**
				V (17%)			
	37	H	R	H (100%)	H (100%)	H (100%)	H (100%)
NS1	74	D	N	D (99%)	D (100%)	S (85%)	D (100%)
						D (14%)	
						H (1%)	
				A (1%)			
NS2	75	E	V	E (99%)	E(100%)	E(100%)	E(100%)
				K (0.7%)			
				D (0.3%)			

^a^H9 numbering

^b^H3 numbering is in parentheses

^c^The substitutions also occurred in mice-adapted influenza viruses in the previous studies

^d^The substitutions which present more frequently in human influenza viruses than H9N2 AIVs was shown by bold and underline formats

Our results show that the G1 and ZB H9N2 viruses could evolve to be highly pathogenic for mice after serial passage in mouse lungs, and the adaptation of the H9N2 AIVs to mice involves multiple amino acid substitutions in viral polypeptides. Several substitutions were not detected in the mice-adapted strains previously and many of them showed higher frequency in human viruses than H9N2 AIVs, indicating their importance for mammalian adaptation. Previous studies reported that influenza viruses can cause not only respiratory diseases but also nervous system syndrome [19] and diseases of the nervous system could pose a serious threat to the host health. Clinical studies showed that 9.7% of the children infected with pH1N1/2009 influenza virus developed central nervous system injury and nervous system syndrome, including hyperthermia, encephalitis, and acute necrotic encephalopathy, which increased the mortality of the patients [20]. H5N1 was isolated from the cerebrospinal fluid of a boy virus enters the CNS through the nerve pathways [21], and virus antigens and RNA can be detected the in sympathetic ganglion in mice. The ZB-MA virus becomes neurovirulent in mammals, which is not observed in the H9N2 mice-adapted strains previously and considered to be one of the main factors leading to the fatal course of infection in humans. Additionally, G1-MA might also acquire such ability after more passages. As we all know, H5 and H7 subtypes found in highly pathogenic avian influenza-infected humans have caused severe pneumonia and a very high death rate, particularly H5N1, which can cause severe disease and high mortality. This is because a low pathogenic avian influenza virus usually infects the digestive tract or respiratory tract of mammals, while the highly pathogenic ones could cause systemic infection (having been isolated from multiple organs). Highly pathogenic avian influenza viruses cause damage to cells mainly through cell necrosis or apoptosis. High levels of viral replication are associated with the extent of necrosis, which usually results in large amounts of AIVs NP proteins detected in the nuclei and cytoplasm of infected cells [22–24]. Therefore, we should pay close attention to the evolution of H9N2 virus to prevent the emergence of new influenza viruses that are a threat to public health. Collectively, our report established a theoretical framework to study the adaptation and epidemic potential of H9N2 AIVs in mammals.

## Data Availability

The datasets used and/or analysed during the current study are available from the corresponding author on reasonable request.

## Abbreviations

A: Alanine (Ala)
AIV: Avian influenza virus
C: Cysteine (Cys)
CNS: Central nervous system
D: Aspartic acid (Asp)
dpi: days post inoculation
E: Glutamic acid (Glu)
F: Phenylalanine (Phe)
G: Glicine (Gly)
H: Histidine (His)
HA: Hemagglutinin
I: Isoleucine (Ile)
K: Lysine (Lys)
L: Leucine (Leu)
M: Matrix proteins
M: Methionine (Met)
MLD_50_: 50% mouse lethal dose
N: Asparagine (Asn)
NA: Neuraminidase
NP: Nucleoprotein
NS: Nonstructural protein
P: Proline (Pro)
PA: Polymerase acidic protein
PB1: Polymerase basic protein 1
PB2: Polymerase basic protein 2
pfu: Plaque-forming unit
Q: Glutamine (Gln)
R: Arginine (Arg)
RNA: Ribonucleic acid
RNP: RNA-protein complexes
S: Serine (Ser)
T: Threonine (Thr)
V: Valine (Val)
Y: Tyrosine (Tyr)
μL: Microliter
